# Supplementary material for: Comparative neurofilament light chain trajectories in CSF and plasma in autosomal dominant Alzheimer’s disease
Source: Nat Commun. 2024 Nov 18;15:9982. doi: 10.1038/s41467-024-52937-8 (PMC11574007; doi:10.1038/s41467-024-52937-8)
Supplement: Supplementary file 1 — Supplementary Information [file 41467_2024_52937_MOESM1_ESM.pdf]

Supplementary Tables and Figures

Supplementary Table 1. Background characteristics of longitudinal sample.

**Variable**	**NC**, N = 88[1]	**MC**, N = 146[1]	**p-value**[2]
**Baseline age (yrs)**	38.5 (10.8)	41.7 (10.5)	0.048
**Sex**			0.842
Female	50 (57%)	81 (55%)	
Male	38 (43%)	65 (45%)	
**EYO (yrs)**	-9.1 (10.6)	-5.0 (10.4)	0.004
**Baseline BMI**	28.0 (6.2)	27.5 (6.1)	0.459
**Baseline MMSE**	30.1 (7.3)	25.2 (6.7)	9.98e-09
**Family Mutation**			0.611
APP	14 (16%)	27 (18%)	
PSEN1	65 (74%)	109 (75%)	
PSEN2	9 (10%)	10 (6.8%)	
**Baseline precuneus volume (mm3)**	9,493.7 (1,085.4)	8,828.7 (1,722.0)	0.003
**Baseline precuneus PiB-PET (SUVR)**	1.2 (0.4)	2.9 (1.4)	2.019e-16
**Baseline plasma NfL (pg/ml)**	5.5 (2.5)	11.8 (10.9)	5.678e-11
**Baseline CSF NfL (pg/ml)**	239.3 (144.3)	645.0 (631.3)	2.472e-10
**Baseline CDR Global**			2.951e-12
CDR 0	88 (100%)	82 (56%)	
CDR 0.5	0 (0%)	29 (20%)	
CDR 1+	0 (0%)	35 (24%)	

[1]Mean (SD); n (%)

[2]Wilcoxon rank sum test (two-sided); Pearson's Chi-squared test (two-sided)

MC = Mutation carrier; NC = Non-carrier family member; EYO = estimated years from symptom onset; MMSE = Mini Mental Status Examination; BMI = Body mass index; CDR = Clinical Dementia Rating. The overall N is 88 for NC group and 146 for MC, except for precuneus volume (NC = 78 and MC = 127) and precuneus PiB-PET (NC = 61 and MC = 94). Note that not all parameters could be collected from each participant. Sex was self-reported as male or female.

Supplementary Table 2. Full models examining background characteristics and baseline log_{10}NfL.

		Baseline log_{10} Plasma NfL		Baseline log_{10} CSF NfL	
**Mutation group**	**Variable**	**B (SE)**	**p-value**	**B (SE)**	**p-value**
Non-Carrier	Age	0.012 (0.001)	1.72e-16	0.017 (0.001)	8.40e-27
	BMI	-0.010 (0.002)	7.17e-5	-0.003 (0.003)	0.247
	Sex	-0.014 (0.027)	0.606	0.085 (0.028)	0.003
Mutation Carrier	Age	0.016 (0.001)	7.43e-32	0.021 (0.002)	8.18e-33
	BMI	-0.008 (0.002)	4.66e-04	-0.002 (0.003)	0.582
	Sex	-0.023 (0.025)	0.365	0.088 (0.033)	0.008

Unstandardized beta estimates, standard error (SE), and corresponding p-values from linear regression models examining association of age, body mass index (BMI), and sex on cross-sectional cerebrospinal fluid (CSF) and plasma neurofilament light chain (NfL) in mutation carriers and non-carriers. Self-reported female sex is the reference group.

Supplementary Table 3. Covariate-adjusted association between log_{10}plasma and log_{10}CSF NfL.

		Baseline log_{10} Plasma NfL		Δ log_{10} Plasma NfL	
**Mutation group**	**Variable**	**B (SE)**	**p-value**	**B (SE)**	**p-value**
Non-Carrier	Baseline Age	0.007 (0.002)	1.67e-04	0.0001 (<0.001)	0.035
	Baseline BMI	-0.009 (0.002)	1.53e-04	-0.0004 (<0.001)	7.24e-05
	Sex	-0.038 (0.026)	0.148	-0.002 (0.001)	0.091
	Baseline log_{10} CSF NfL	0.288 (0.073)	1.19e-04	NA	NA
	Δlog_{10} CSF NfL	NA	NA	0.375 (0.091)	9.45e-05
Mutation Carrier	Baseline Age	0.003 (0.001)	7.05e-04	0.0002 (<0.001)	0.007
	Baseline BMI	-0.007 (0.001)	1.63e-06	-0.0004 (<0.001)	7.64e-05
	Sex	-0.076 (0.016)	5.84e-06	-0.004 (0.001)	3.48e-04
	Baseline log_{10} CSF NfL	0.596 (0.030)	3.72e-55	NA	NA
	Δlog_{10} CSF NfL	NA	NA	0.067 (0.055)	4.57e-24

Unstandardized beta estimates, standard error (SE), and corresponding p-values from linear regression and linear mixed effects models comparing cross-sectional and longitudinal cerebrospinal fluid (CSF) and plasma neurofilament light chain (NfL) in mutation carriers and non-carriers, adjusting for age, body mass index (BMI), and sex. Self-reported female sex is the reference group.

Supplementary Table 4. Association between rate of change in NfL and AD clinical groupings.

Comparison	B	SE	p-value
	**Δ log_{10}(plasma NfL)**		
Presym vs. NC	0.004	0.002	0.038
Conv vs. NC	0.017	0.003	1.28e-05
Sym vs. NC	0.020	0.002	< 1.00e-90
Conv vs. Presym	0.012	0.003	0.001
Sym vs. Presym	0.015	0.002	1.62e-11
Sym vs. Conv	0.003	0.003	0.772
	**Δ log_{10}(CSF NfL)**		
Presym vs. NC	0.002	0.001	0.096
Conv vs. NC	0.012	0.002	9.33e-08
Sym vs. NC	0.019	0.001	< 1.00e-90
Conv vs. Presym	0.010	0.002	3.65e-05
Sym vs. Presym	0.017	0.001	< 1.00e-90
Sym vs. Conv	0.007	0.002	0.006
	**Plasma/CSF NfL**		
Presym vs. NC	- 0.002	0.001	0.802
Conv vs. NC	- 0.004	0.003	0.509
Sym vs. NC	- 0.010	0.002	8.39e-10
Conv vs. Presym	- 0.003	0.003	0.753
Sym vs. Presym	- 0.009	0.002	6.45e-08
Sym vs. Conv	- 0.006	0.003	0.205

Unstandardized beta estimates, standard error, and corresponding p-values from linear regression and linear mixed models examining association between mutation/cognitive groupings and rate of change in NfL. Presym = Presymptomatic Mutation Carrier (MC) group; NC = Non-carrier group; Conv = Converter MC group; Sym = Symptomatic MC group.

Suplementary Table 5. Longitudinal association between NfL and AD biomarkers.

Cognitive Group	Δ log_{10} Plasma NfL				Δ log_{10} CSF NfL			
	Δ Amyloid - PET		Δ Precuneus Volume		Δ Amyloid - PET		Δ Precuneus Volume	
	B(SE)	p	B(SE)	p	B(SE)	p	B(SE)	p
**NC**	-0.048 (0.4)	0.912	265 (873)	0.752	-0.737 (1.3)	0.568	-884 (2,630)	0.740
**PreSym**	1.916 (1.2)	0.127	-1,154 (1,105)	0.302	0.130 (3.6)	0.971	-1,143 (2,618)	0.667
**Sym**	0.617 (1.4)	0.672	−7,193 (1,947)	8.73e-04	1.003 (0.003)	0.871	−23,658 (5,537)	1.20e-04

Unstandardized beta estimates (B), standard errors (SE), and multiple comparison corrected exact p-values (p) for Δ log_{10} NfL*time term from linear mixed effects models adjusted for baseline age*time, baseline BMI*time, and sex*time. Presym = Presymptomatic Mutation Carrier (MC) group; NC = Non-carrier group; Conv = Converter MC group; Sym = Symptomatic MC group.

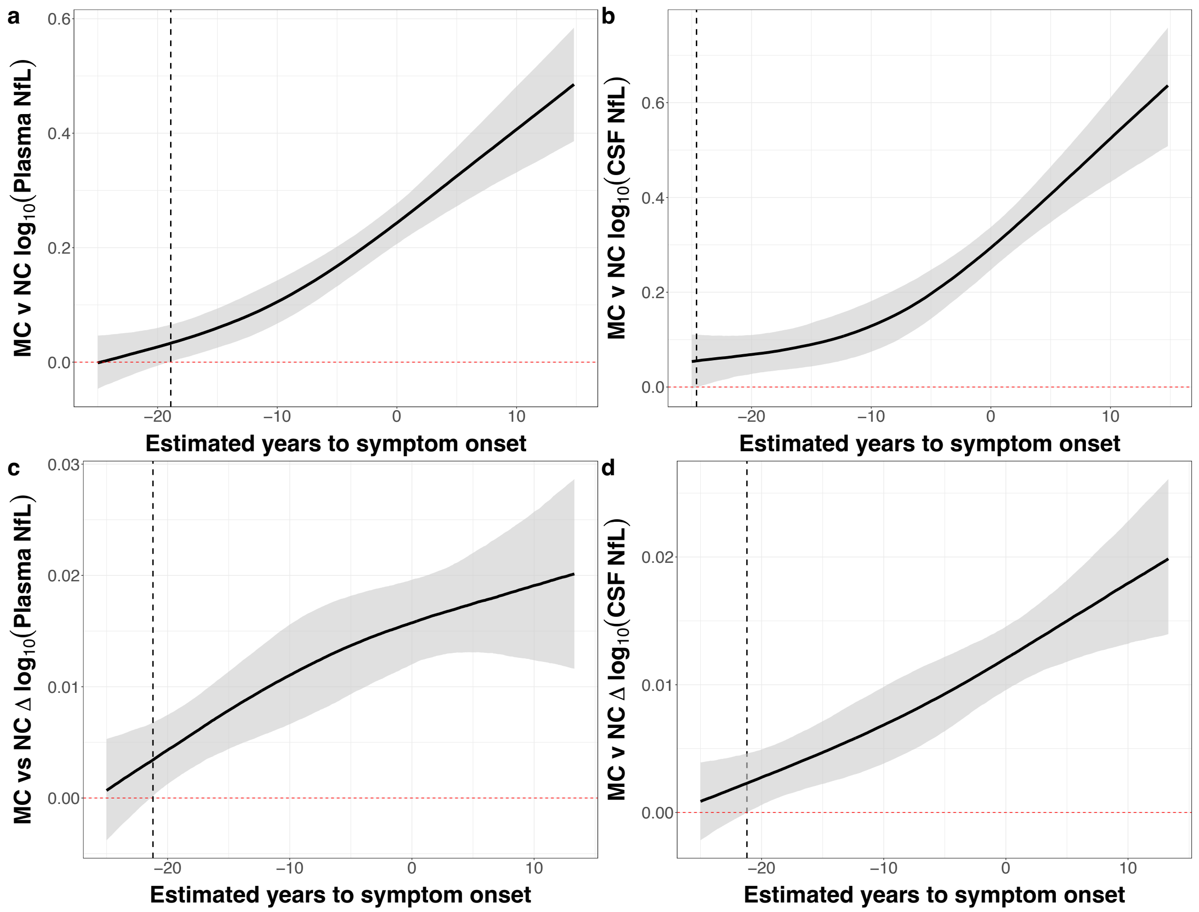

Supplementary Fig. 1: Difference distribution curves for cross-sectional and longitudinal plasma and CSF NfL levels in mutation carriers and non-carriers. Difference of posterior distribution for cross-sectional (**a**) plasma and (**b**) CSF log10(NfL) and longitudinal (**c**) plasma and (**d**) CSF log10(NfL) between mutation carriers (MC) and non-carriers (NC), as a function of Estimated years of onset (EYO). The solid black line depicts the median of the difference distribution and the shaded area represents the 95% equal-tailed credible intervals. EYO was considered statistically significant if the 95% equal-tailed credible intervals of the posterior distribution did not overlap 0. This EYO where MC and NC groups first diverge is represented by a vertical dashed line.

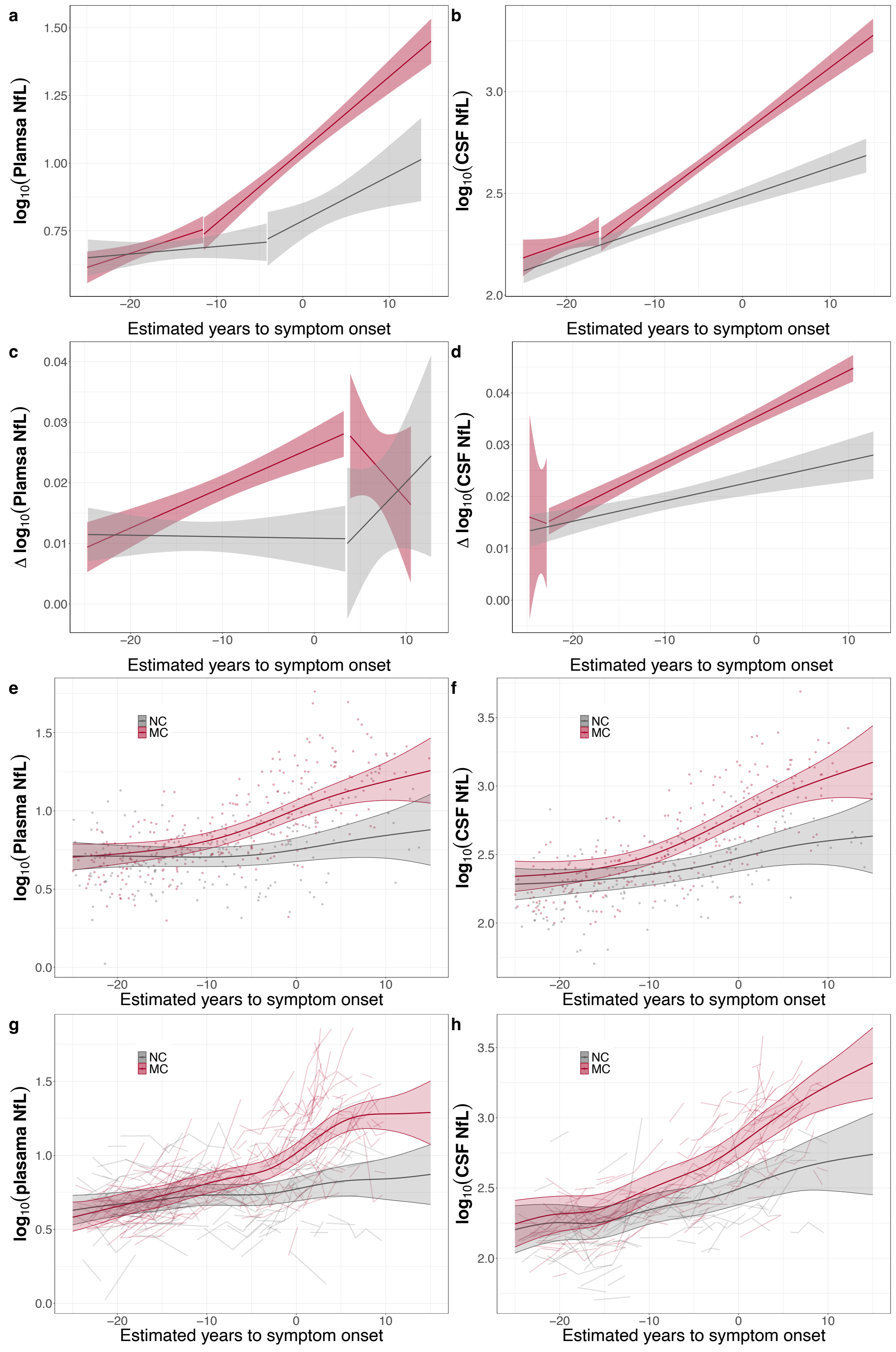

Supplementary Fig. 2: Piecewise regression analyses for cross-sectional and longitudinal plasma and CSF NfL levels in mutation carriers and non-carriers. A piecewise regression analysis was performed for baseline **(a)** plasma and **(b)** CSF log10(NfL) and longitudinal rate of change in (**c**) plasma and **(d)** CSF log10(NfL) as a function of EYO in mutation carriers (MC, red line) and non-carriers (NC, grey line). Bifurcation point was determined with the segmented package in R. Individual points showing cross-sectional (**e,f**) and spaghetti plots showing longitudinal (**g,h**) plasma and CSF log10(NfL) for NC (grey) and MC (red). Spaghetti plots are restricted to EYOs between -20 and +10. The curves are generated from spline-based confidence intervals from fitted generalised additive models (GAMs).
