# Supplementary material for: Comparative neurofilament light chain trajectories in CSF and plasma in autosomal dominant Alzheimer’s disease
Source: Nat Commun. 2024 Nov 18;15:9982. doi: 10.1038/s41467-024-52937-8 (PMC11574007; doi:10.1038/s41467-024-52937-8)
Supplement: Supplementary file 3 — Reporting Summary [file 41467_2024_52937_MOESM3_ESM.pdf]

nature portfolio

Corresponding author(s): Mathias Jucker and Stephanie Schultz

Last updated by author(s): 7/30/2024

Reporting Summary

Nature Portfolio wishes to improve the reproducibility of the work that we publish. This form provides structure for consistency and transparency in reporting. For further information on Nature Portfolio policies, see our Editorial Policies and the Editorial Policy Checklist.

Statistics

For all statistical analyses, confirm that the following items are present in the figure legend, table legend, main text, or Methods section.

n/a	Confirmed	
☐	☒	The exact sample size (*n*) for each experimental group/condition, given as a discrete number and unit of measurement
☐	☒	A statement on whether measurements were taken from distinct samples or whether the same sample was measured repeatedly
☐	☒	The statistical test(s) used AND whether they are one- or two-sided<br>*Only common tests should be described solely by name; describe more complex techniques in the Methods section.*
☐	☒	A description of all covariates tested
☐	☒	A description of any assumptions or corrections, such as tests of normality and adjustment for multiple comparisons
☐	☒	A full description of the statistical parameters including central tendency (e.g. means) or other basic estimates (e.g. regression coefficient) AND variation (e.g. standard deviation) or associated estimates of uncertainty (e.g. confidence intervals)
☐	☒	For null hypothesis testing, the test statistic (e.g. F, t, r) with confidence intervals, effect sizes, degrees of freedom and P value noted<br>*Give P values as exact values whenever suitable.*
☐	☒	For Bayesian analysis, information on the choice of priors and Markov chain Monte Carlo settings
☐	☒	For hierarchical and complex designs, identification of the appropriate level for tests and full reporting of outcomes
☐	☒	Estimates of effect sizes (e.g. Cohen's *d*, Pearson's *r*), indicating how they were calculated

Our web collection on statistics for biologists contains articles on many of the points above.

Software and code

Policy information about availability of computer code

Data collection	No software was used.
Data analysis	All analyses were performed in R . Models were fitted using lme4 in R. The unstandardized regression coefficients (B), standard error of the mean (s.e.m.), and P values from the LMMs and linear regression models are reported in the supplemental tables. source code available, with publication, at https://github.com/stephaschultz/DIAN_plasma_CSF_NfL.

For manuscripts utilizing custom algorithms or software that are central to the research but not yet described in published literature, software must be made available to editors and reviewers. We strongly encourage code deposition in a community repository (e.g. GitHub). See the Nature Portfolio guidelines for submitting code & software for further information.

Data

Policy information about availability of data

All manuscripts must include a data availability statement. This statement should provide the following information, where applicable:

- Accession codes, unique identifiers, or web links for publicly available datasets
- A description of any restrictions on data availability
- For clinical datasets or third party data, please ensure that the statement adheres to our policy

Individual-level data from the Dominantly Inherited Alzheimer's Network observational study (DIAN-OBS) cannot be shared publicly owing to the need for participant anonymity. However, DIAN-OBS data included in this analysis can be accessed by qualified researchers upon request submitted at https://dian.wustl.edu/our-research/for-investigators/dian-observational-study-investigator-resources/data-request-form/.

Research involving human participants, their data, or biological material

Policy information about studies with human participants or human data. See also policy information about sex, gender (identity/presentation), and sexual orientation and race, ethnicity and racism.

Reporting on sex and gender	In our demographic breakdown of the DIAN-OBS data, we have included percentage of sample reported as female and males to describe the distributions of biological sex within our cohorts. Sex was considered and included as a covariate in all primary and exploratory models.
Reporting on race, ethnicity, or other socially relevant groupings	n.a.
Population characteristics	Plase see also table 1 of our manuscript. The covariate relevant characteristics of our cohort were the following: age, sex and BMI.
Recruitment	Participants were recruited through the various DIAN collaboration sites, as well as through broader efforts such as: http://dian-info.org/, http://www.alzforum.org/new/detail.asp?id=1967, http://www.alz.org/trialmatch and http://www.dianexpandedregistry.org/. As individuals come from families with known histories of ADAD, there is a strong possibility for selection bias, or prior knowledge of ADAD upon entering the study. To remove some forms of this bias, participants are not required to learn their mutation carrying status, and study coordinators running assessments are blinded to their mutation carrying and CDR status at time of testing.
Ethics oversight	The institutional review board at Washington University in St. Louis provided supervisory review and human studies approval. Each site's institutional review board approved all study procedures. Participants or their caregivers provided informed consent in accordance with their local institutional review boards. Ethics committee at the medical faculty of the University of Tübingen, Germany (project number 718/2014BO2).

Note that full information on the approval of the study protocol must also be provided in the manuscript.

Field-specific reporting

Please select the one below that is the best fit for your research. If you are not sure, read the appropriate sections before making your selection.

☒ Life sciences ☐ Behavioural & social sciences ☐ Ecological, evolutionary & environmental sciences

For a reference copy of the document with all sections, see nature.com/documents/nr-reporting-summary-flat.pdf

Life sciences study design

All studies must disclose on these points even when the disclosure is negative.

Sample size	We used all data available within the DIAN data freeze version no. 15, i.e. n=567 individuals.
Data exclusions	Data from NC with an EYO > 15 years were excluded as there were no corresponding MC with data in this later EYO range and showing these data have the potential for unblinding. Reverters (those who were CDR > 0 at a visit and CDR = 0 at any subsequent visit) were excluded from analyses, as this clinical situation is inconclusive. Data from NC with a CDR >0 (i.e., 0.5 in all the cases, n=14), either within all visits available or at least the latest visit were excluded, as they probably have a reason for cognitive cedline different from AD. Individuals with a Dutch-type CAA pathogenic variant (APP E693Q; n = 10 MC and n = 13 NC family members) or variants believed to be non-pathogenic or weakly pathogenic (n= 13 and n = 5 NC family members) were excluded from this study, as they probably do not have the typical AD phenotype. Previous medical histories revealed that three individuals with inconclusively high NfL values had competing neurological disorders and were excluded from analyses (to maintain blinding the specific casues are not mentioned here).
Replication	All CSF and plasma samples were measured in duplicates. We will provide code publicly that can be used by researchers who request DIAN-OBS data to replicate the analyses described within. This code will also allow researchers to alter data analyses to match their individual project needs.
Randomization	There were no experimental groups within our study.

Blinding	Those who were directly testing the individuals remained blinded to their impairment and mutation-carrying status.

Reporting for specific materials, systems and methods

We require information from authors about some types of materials, experimental systems and methods used in many studies. Here, indicate whether each material, system or method listed is relevant to your study. If you are not sure if a list item applies to your research, read the appropriate section before selecting a response.

Materials & experimental systems

n/a	Involved in the study
☐	☒ Antibodies
☒	☐ Eukaryotic cell lines
☒	☐ Palaeontology and archaeology
☒	☐ Animals and other organisms
☐	☒ Clinical data
☒	☐ Dual use research of concern
☒	☐ Plants

Methods

n/a	Involved in the study
☒	☐ ChIP-seq
☒	☐ Flow cytometry
☐	☒ MRI-based neuroimaging

Antibodies

Antibodies used	Measurements were performed on a Single molecule array platform (Simoa, HD-X analyzer; Quanterix) with commercially available assay kits (NF-Light Advantage Kit Cat 103186).
Validation	These were ready-to-use and already validated antibodies. We mention that Blood and CSF samples were collected and initially processed with the same methods described (Preische et al., 2019).

Clinical data

Policy information about clinical studies

All manuscripts should comply with the ICMJEguidelines for publication of clinical research and a completedCONSORT checklist must be included with all submissions.

Clinical trial registration	The Dominantly Inherited Alzheimer Network (DIAN, U19AG032438).
Study protocol	*Note where the full trial protocol can be accessed OR if not available, explain why.*
Data collection	*Describe the settings and locales of data collection, noting the time periods of recruitment and data collection.*
Outcomes	n.a.

Plants

Seed stocks	n.a.
Novel plant genotypes	n.a.
Authentication	n.a.

Magnetic resonance imaging

Experimental design

Design type	Structural MRI for diagnostic purpose.
Design specifications	n.a.
Behavioral performance measures	n.a.

Acquisition

Imaging type(s)	structural
Field strength	3T
Sequence & imaging parameters	T1-weighted imaging to assess the degree of atrophy.
Area of acquisition	Whole brain scan.

Diffusion MRI ☐ Used ☒ Not used

Preprocessing

Preprocessing software	Processing has been performed using FreeSurfer v 5.3 (http://surfer.nmr.mgh.harvard.edu/) and the Desikan-Killany atlas to produce regional estimates of grey matter volume within brain regions. As has been done previously, analyses focused on the precuneus as the a priori region of interest (ROI). Precuneus volumes were adjusted for total intracranial volume prior to statistical analysis.
Normalization	Freesurfer's recon-all flag includes transformation to Talairach space and aseg atlas
Normalization template	Talairach
Noise and artifact removal	Freesurfer's recon-all function employs motion correction, removal of non-brain structures using a watershed deformation procedure, intensity normalization, these specific descriptions within the manuscript include citations. Furthermore, quality control technicians screen images prior to preprocessing to ensure any major deviations or artifacts are caught and they will request sites to re-scan individuals if necessary. Finally, further quality control procedures occur at the end of the Freesurfer recon-all preprocessing to ensure remaining artifacts or errors are removed. These processing technicians will run error checks and subsequent reprocessing of the data up to three times before images are considered to have failed.
Volume censoring	We do not censor volumes, we only remove data from the data release that have freesurfer errors that cannot be rectified.

Statistical modeling & inference

Model type and settings	Separate models were run for each NC, all MC, pre-symptomatic MC, and symptomatic MC groups. The dependent term for each model was a time-varying imaging biomarker with fixed effect terms for baseline age*time, sex*time, baseline BMI*time, and interaction between extracted rate of change in CSF or plasma NfL and time. Models contained random slope and intercept terms for participants and random intercepts for family. The primary term of interest was the interaction between the rate of change in CSF or plasma NfL and time. Models were fitted using lme4 in R. The unstandardized regression coefficients (B), standard error of the mean (s.e.m.), and P values from the LMMs and linear regression models are reported in the supplementary tables.
Effect(s) tested	Please see above.

Specify type of analysis: ☐ Whole brain ☒ ROI-based ☐ Both

Anatomical location(s): Precuneus, please see above.

Statistic type for inference (See Eklund et al. 2016)	Extracted averages are used for these analyses.
Correction	Please see above.

Models & analysis

n/a	Involved in the study
☒	☐ Functional and/or effective connectivity
☒	☐ Graph analysis
☐	☒ Multivariate modeling or predictive analysis

Multivariate modeling and predictive analysis	Separate models were run for each NC, all MC, pre-symptomatic MC, and symptomatic MC groups. The dependent term for each model was a time-varying imaging biomarker with fixed effect terms for baseline age*time, sex*time, baseline BMI*time, and interaction between extracted rate of change in CSF or plasma NfL and time. Models contained random slope and intercept terms for participants and random intercepts for family. The primary term of interest was the interaction between the rate of change in CSF or plasma NfL and time. Models were fitted using lme4 in R. The unstandardized regression coefficients (B), standard error of the mean (s.e.m.), and P values from the LMMs and linear regression models are reported in the supplementary tables.
